# Dietary intake patterns and nutritional adequacy among adults with overweight or obesity treated with GLP-1 or dual GIP/GLP-1 receptor agonists- preliminary study

**DOI:** 10.1186/s12967-026-07702-4

**Published:** 2026-04-11

**Authors:** Sebastian Korus, Danuta Cembrowska-Lech, Karolina Kłoda, Ewa Stachowska

**Affiliations:** 1 Department of Human Nutrition and Metabolomics, Pomeranian Medical University, Szczecin, Poland; 2 Sanprobi Sp. z o. o. sp. k., Szczecin, PL-70-535 Poland; 3 Centre for Molecular Biology and Biotechnology, Institute of Biology, University of Szczecin, Szczecin, PL-71-415 Poland; 4 Polish Society of Family Medicine and Polish Society of Obesity Treatment, Poznan, Poland

## Abstract

**Background:**

GLP-1 dual GIP/GLP-1 agonists significantly suppress appetite, but it is unclear whether the typical diet of patients treated with these drugs still meets their basic nutritional need.

**Objective:**

To assess nutrients intake among adults undergoing pharmacotherapy and identify dietary predictors of weight loss efficacy.

**Methods:**

This retrospective cohort study was conducted online among social media support groups (April 2024–February 2025). Participants included 387 adults, who reported regular once-weekly use of a GLP-1 agonist or dual GIP/GLP-1 agonist and completed 48-hour food diaries (one weekday and one weekend day). Daily energy and nutrient intake were reconstructed using a specialized software (Diet 6) containing an up-to-date database and standards issued by the National Institute of Public Health (NIZP PZH – PIB 2024). Differences between week and weekends were analyzed using a paired-samples t-test. Multiple linear regression assessed dietary and treatment predictors of weight loss.

**Results:**

Average energy intake was 753 kcal (SD 257.8 kcal), with protein 33.4 g (SD 15.3 g), fat 26.5 g (SD 12.4 g), carbohydrates 96.4 g (SD 35.6 g), and fiber 7.2 g (SD 3.1 g). Less than 10% of participants met the recommended intakes for protein. Weekday intake was significantly higher by 170 kcal (95%CI 152–185 kcal; *p* < 0.001), with greater consumption of fat (8–9 g), and sodium (370 mg). Higher total protein intake was “associated with more weight loss” (β = 0.446; *p* = 0.014), while a higher proportion of animal protein (β = − 0.517; *p* = 0.004) and higher sodium intake (β ≈ − 0.002; *p* = 0.013) were associated with less weight loss.

**Conclusions:**

Adults receiving GLP-1 pharmacotherapy exhibit insufficient protein and critical micronutrients, alongside excess fat and sodium intake on weekdays. Routine dietary education emphasizing protein adequacy and micronutrient sufficiency should accompany incretin therapy to prevent nutritional deficiencies, sarcopenia, and optimize weight loss outcomes.

## Introduction

Glucagon-like peptide-1 (GLP-1) receptor agonists and dual Gastric inhibitory polypeptide (GIP) and glucagon-like peptide‐1 (GLP‐1)GIP/GLP-1 agonists are currently the most effective pharmacotherapy for obesity and type 2 diabetes [1–5]. By delaying gastric emptying and increasing satiety, as well as by influencing the hunger and satiety centers in the hypothalamus and the reward system, these drugs enable significant weight loss and improve cardiovascular and metabolic risk [6–12] 1 However, the same mechanisms that promote energy deficit can lead to chronic protein and micronutrient deficiencies, increasing the risk of sarcopenia, impaired wound healing, and reduced immunity [13–15]. It is estimated that 9–12 million people worldwide are currently using GLP-1-based therapy, but their usual diet has not yet been systematically assessed [16].

Previous studies have focused mainly on weight loss and glycemic control; few describe the macronutrient and micronutrient profile during therapy, and none compare intake on weekdays and weekends [17–19]. There is also a lack of data on whether treatment duration, drug class (GLP-1 vs. GIP/GLP-1), or qualitative diet composition modify weight loss efficacy [3, 5, 20]. It is also unclear whether rapid loss of lean body mass (which is an independent risk factor for sarcopenia, gallstones, and recurrence of metabolic disorders) may increase the risk of regaining weight after discontinuation of the drug [21–25]. 

The aim of this study was to assess energy intake and macro- and micronutrient intake on weekdays and weekends in adults reporting regular use of GLP-1 or GIP/GLP-1 agonists. The hypotheses were that (1) most patients do not meet the recommended intake standards for protein and key micronutrients, and (2) higher total protein intake, but not a predominance of animal protein, independently predicts greater weight loss, after adjusting for drug class and duration of therapy.

## Methods

### Study design

The study was designed as a **retrospective observational cohort** conducted exclusively online, in accordance with the STROBE guidelines for observational epidemiological studies. The analysis includes users of GLP-1 receptor agonists (dulaglutide, liraglutide, semaglutide) or dual GIP/GLP-1 agonists (tirzepatide) recruited from Polish online communities. All participants provided their informed consent electronically.

Ethics and Consent to Participate declarations: not applicable.

### Data source

The data was collected using a Google Forms form hosted on a secure researcher account with two-factor authentication. The anonymized data sets were stored and analyzed exclusively locally; the identifiers corresponded to random codes assigned to the respondents, which complies with the requirements of the The General Data Protection Regulation (GDPR). Participants were recruited between April 2024 and February 2025. Recruitment channels included targeted posts in Polish-language support groups on Facebook™ and Instagram™ for people with obesity and/or diabetes using incretins. The inclusion criteria for the study were: age ≥ 18 years, current use of GLP-1 or GLP-1 and GIP analogues, pharmacological treatment for obesity or metabolic disorders, and informed consent to participate in the study. Minors, individuals not taking the listed medications, or those who did not complete the questionnaire were excluded from participation. All participants provided their informed consent electronically.

Ethics and Consent to Participate declarations: not applicable.

Anthropometric data (body weight, height) were collected online and included self-reported body weight before the start of therapy and during the study, along with a complete 48-hour food diary (typical weekday (mostly Fryday and weekend mostly Saturday).

The final sample included 387 adults (80.4% women; mean age 34.9 ± 9.9 years; baseline BMI 36.4 ± 5.0 kg/m²). Types of drugs used by respondents: GLP-1 RA (dulaglutide, liraglutide, semaglutide) vs. dual GIP/GLP-1 agonist (tirzepatide). Average duration of therapy reported by respondents was 15.7 (SD 14 weeks)- Table 1.

The study collected information on dietary profiles. The form consisted of two parts covering demographic data – age, gender, body weight before therapy and current body weight, height, duration of drug use (weeks), type of preparation, and any bariatric surgery.

A 48-hour food diary – weighted, covering one typical working day and one weekend day; respondents recorded the time, weight or household measure, method of preparation, and brand of the product. Before sending the form, the system checked for completeness, and the user could correct any omissions.

Respondents recorded in detail all food items with weighed portions or household measures for two days: a working day and a weekend day. The records were transferred to the Dieta-6 software (Poland, NIZP PHZ-PIB 2024 database), which generated values for energy and 42 nutrients. The results were compared with current Polish nutritional standards. Data were obtained on the average daily intake of macronutrients (using a 48-hour food diary completed by respondents) and key micronutrients (total protein, animal and plant protein, fiber, calcium, potassium, vitamins D, E, C) on weekday and weekend.

The primary endpoints in this study were to estimate the percentage of participants meeting the RDA/AI standards for protein and selected micronutrients, and the difference in energy and macronutrient intake between weekdays and weekends. Secondary endpoints were changes in body weight (kg) and BMI from the start of treatment, as reported by respondents.

The study complies with the principles of the Declaration of Helsinki. Anonymization prevents the identification of individuals, therefore, it was not necessary to obtain additional consent for the disclosure of secondary data. The results concerning dietary adequacy and changes in body weight/BMI were analyzed separately.

### Study population

#### Outcomes

Adequacy of intake included consumption of carbohydrates, total fat, fiber, protein (total and divided into animal/plant sources) and sodium from weekday, weekends, and their average was compared with the Polish Nutrition Standards 2024 (DRI). For each of the five food categories, each observation was classified as deficit – value < lower limit of DRI, balanced – value within DRI, excess – value > upper limit of DRI.

From these classifications, the percentage of participants in each category was calculated for weekday, weekend and the two-day average; these results were visualized to show the degree of compliance with national recommendations.

The primary outcome was the proportion of participants in the “balanced” category for each component at the three measurement points. Secondary outcomes included (a) the difference in energy, macronutrient, and fiber intake between weekdays and weekends;

self-reported change in body weight (kg) and BMI from the start of incretin therapy, calculated using the formula

$$\begin{aligned}&\mathrm{\%}\:\mathrm{d}\mathrm{i}\mathrm{f}\mathrm{f}\mathrm{e}\mathrm{r}\mathrm{e}\mathrm{n}\mathrm{c}\mathrm{e}\hspace{0.17em}=\hspace{0.17em}100\cr & \quad\times\:\:\left(\right(\mathrm{w}\mathrm{e}\mathrm{e}\mathrm{k}\mathrm{e}\mathrm{n}\mathrm{d}\:-\:\mathrm{w}\mathrm{e}\mathrm{e}\mathrm{k}\mathrm{d}\mathrm{a}\mathrm{y})\:/\:\mathrm{w}\mathrm{e}\mathrm{e}\mathrm{k}\mathrm{d}\mathrm{a}\mathrm{y})\end{aligned}$$

The distributions of key variables were assessed visually (histograms, box plots) and based on skewness; variables showing significant asymmetry were log-transformed or standardized. For paired comparisons (weekday vs. weekend), a t-test for dependent samples was used. The relationship between body weight/BMI changes and dietary factors, age, and gender was assessed using an OLS regression model; β coefficients, 95% CI, and p-values were reported; p-value of < 0.05 was considered statistically significant.

**Table 1:** Study population baseline characteristics

Characteristics	Mean or *N* (SD or %) (*N* = 387)
Sex, F / M	311 (80.4%) / 76 (19.6%)
Age, year	34.9 (9.9)
Baseline weight, kg	102.4 (16.9)
Baseline BMI	36.4 (5.0)
Baseline BMI class	
Overweight: 27–30	21 (5.43%)
Obesity class 1 (30 to < 35)	153 (39.53%)
Obesity class 2 (35 to < 40)	139 (35.92%)
Obesity class 3 (≥ 40)	74 (19.12%)
Index medication	387 (100)
Dulaglutide	2 (0.5%)
Liraglutide	88 (22.7%)
Semaglutide	211 (54.5%)
Tirzepatide	86 (22.3%)
Bariatric surgery history	7 (1.8%)

Values are unadjusted means (SD) or n (%). Abbreviations: F, female; M, male; BMI, body mass index

### Statistical analysis

All statistical analyses were performed to evaluate changes between weekday and weekend and to examine how dietary intake related to observed differences in body weight and BMI. For each participant, absolute and percentage changes were computed, with percentage change defined as 100 × ((weekend – weekday)/ weekday). Data distributions for body weight, macronutrient intake, and their day-to-day changes were assessed visually and with the Shapiro–Wilk test. Normally distributed variables were compared using paired t-tests, whereas non-normally distributed variables were evaluated with the Wilcoxon signed-rank test.

Associations between weight or BMI changes and dietary intake were further explored using ordinary least squares regression. Models included absolute changes in macronutrients (protein, fat, carbohydrate, fiber, and sodium), age, sex, treatment time, and receptor agonist use as covariates. In sensitivity analyses, macronutrient intake was expressed as percentage of total energy. Regression coefficients (β) with 95% CIs are reported.

To contextualize dietary patterns, intakes of macronutrients and sodium were compared against the Polish Dietary Reference Intakes and classified as below, within, or above the recommended ranges. The proportions of participants in each category were calculated for weekday, weekend, and the average of both days.

All analyses were conducted using Python (v3.10; statsmodels package). Statistical significance was set at *P* < 0.05 (2-sided).

## Results

### Dietary intake patterns compared with Polish dietary reference intakes

**Fig. 1:**
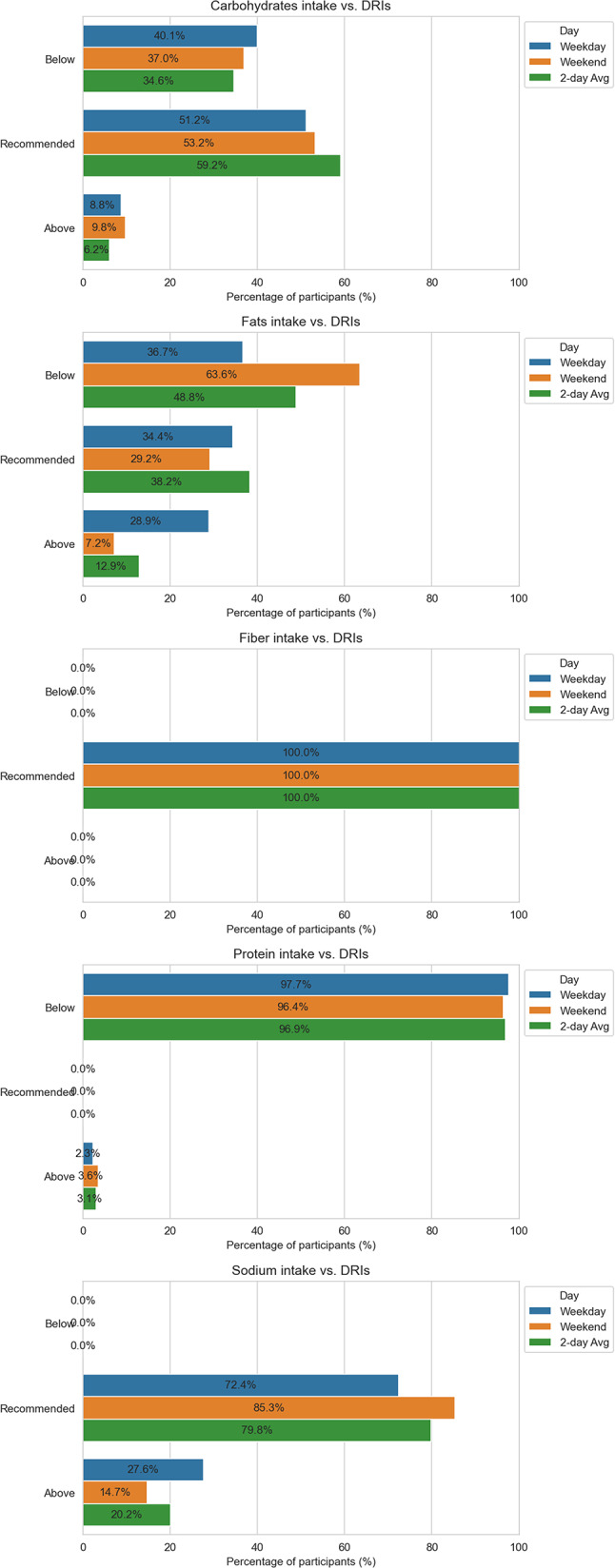
Distribution of carbohydrate, fat, protein, fiber, and sodium intakes relative to Dietary Reference Intakes (DRI) across two 24-h dietary records. Bars indicate the percentage of participants below, meeting, or above the reference value (*n* = 387)

Figure 1 shows the distribution of carbohydrate, fat, protein, fiber, and sodium intakes relative to Dietary Reference Intakes across two 24-h dietary records. Analysis of two-day dietary records showed that approximately half of participants consumed carbohydrates within the recommended range on both days (51–53%) and when averaged across days (59%). About one-third consumed less than recommended, while 6–10% exceeded the upper limit.

Fat intake varied between days. On weekday, participants were evenly distributed across categories of below (37%), within (34%), and above (29%) the Adequate Intake (AI). By weekend, nearly two-thirds consumed less than the AI, < 30% met the guideline, and about 7% exceeded it. Based on the two-day average, 38% met the recommendation, 49% consumed less, and 13% exceeded it.

Fiber intake was markedly adequate. The mean intake was 25 g/day, and all participant met the recommendation.

Protein intake was substantially below the Recommended Dietary Allowance (RDA). Across both days, about 97% failed to meet the requirement, while only 2–4% exceeded it. None met the exact RDA.

Sodium intake was more aligned with recommendations: 72–85% of participants were at or near the AI, while 15–28% exceeded it; no participant consumed less than the guideline.

To sum up, this profile points to a carbohydrate-adequate but protein-poor diet with unstable fat intake, highlighting protein (and, secondarily, fat) as primary targets for dietary improvement.

### Within-week day-to-day variability in macronutrient intake

**Fig. 2:**
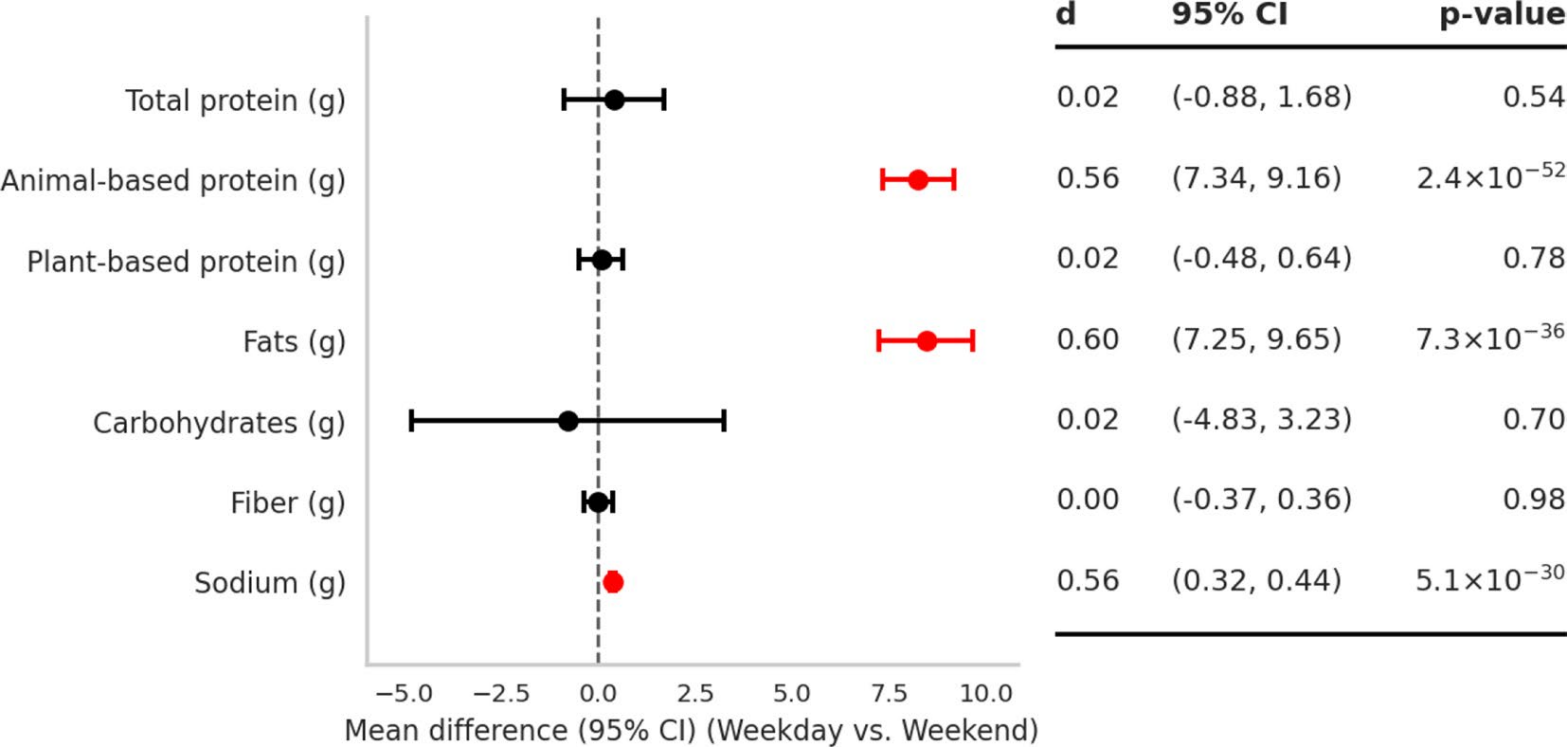
Mean differences in macronutrient intake between weekday and weekends. Points show mean differences with 95% CIs from paired t-tests; the table reports Cohen’s d and p-values (*n* = 387). Positive values indicate higher intake on weekday

Paired analyses compared nutrient intake between weekday and weekend of the final intervention week. Across the two (consecutive or subsequent) days recall days in the week, most macronutrient intakes were stable (Fig. 2). For protein, total intake did not differ between days (mean difference = 0.02 g; 95% CI: − 0.88, 1.68; *P* = 0.54). Similarly, no differences were observed for plant-based protein (0.02 g; 95% CI: − 0.48, 0.64; *P* = 0.78). By contrast, animal-based protein intake was significantly higher on weekday than on weekend (0.56 g; 95% CI: 7.34, 9.16; *P* < 0.001). Fat intake also differed between days, with participants consuming 7–10 g more on weekday than on weekend (0.60 g; 95% CI: 7.25, 9.64; *P* < 0.001). In contrast, carbohydrate and fiber intakes were stable across days, with no significant mean differences [carbohydrates: 0.02 g; 95% CI: − 4.83, 3.23; *P* = 0.71; fiber: 0.01 g; 95% CI: − 0.37, 0.36; *P* = 0.98]. Sodium intake was substantially higher on weekday, averaging about 0.37 g more than on weekend (0.56; 95% CI: 0.32, 0.44; *P* < 0.001).

Taken together, these results indicate that the day-to-day variation in protein intake reflected shifts in protein sources rather than total protein, and that higher weekday sodium accompanied higher fat intake.

### Day-to-day differences in energy intake and macronutrient distribution

**Fig. 3:**
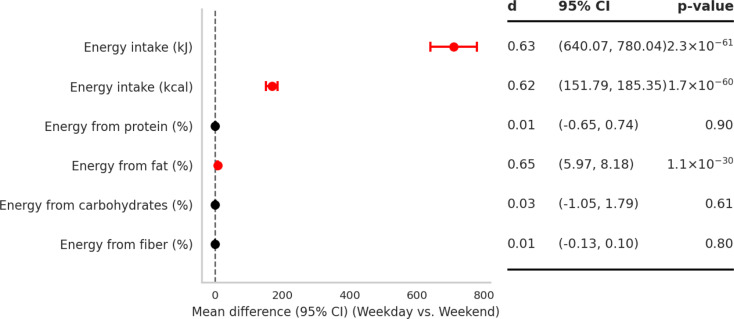
Mean differences in total energy intake and percentage of energy from macronutrients between weekday and weekend of the final intervention week. Points denote mean differences with 95% CIs from paired t-tests (*n* = 387); positive values indicate higher intake or distribution on weekday

As shown in Fig. 3, participants consumed significantly more total energy on weekday compared with weekend, whereas the proportion of energy from protein, carbohydrate, and fiber did not differ between days. Paired analyses showed that total energy intake was significantly higher on weekday compared with weekend (mean difference, 95% CI: 640–780 kJ; *P* < 0.001), equivalent to ~ 170 kcal (95% CI: 152, 185; *P* < 0.001). When examining the proportion of energy from macronutrients, no differences were observed for protein (mean difference: 0.01%; 95% CI: − 0.65, 0.74; *P* = 0.90), carbohydrates (–0.03%; 95% CI: − 1.05, 1.79; *P* = 0.61), or fiber (0.01%; 95% CI: − 0.13, 0.10; *P* = 0.81). In contrast, fat contributed a significantly greater share of total energy intake on weekday compared with weekend (mean difference: 7.1% points; 95% CI: 5.97, 8.18; *P* < 0.001).

Taken together, higher weekday energy intake was accompanied by a higher fat contribution, with no meaningful shifts in the relative contributions of protein, carbohydrates, or fiber.

### Macronutrient intake differences between GLP-1 and dual GIP/GLP-1 users

**Fig. 4:**
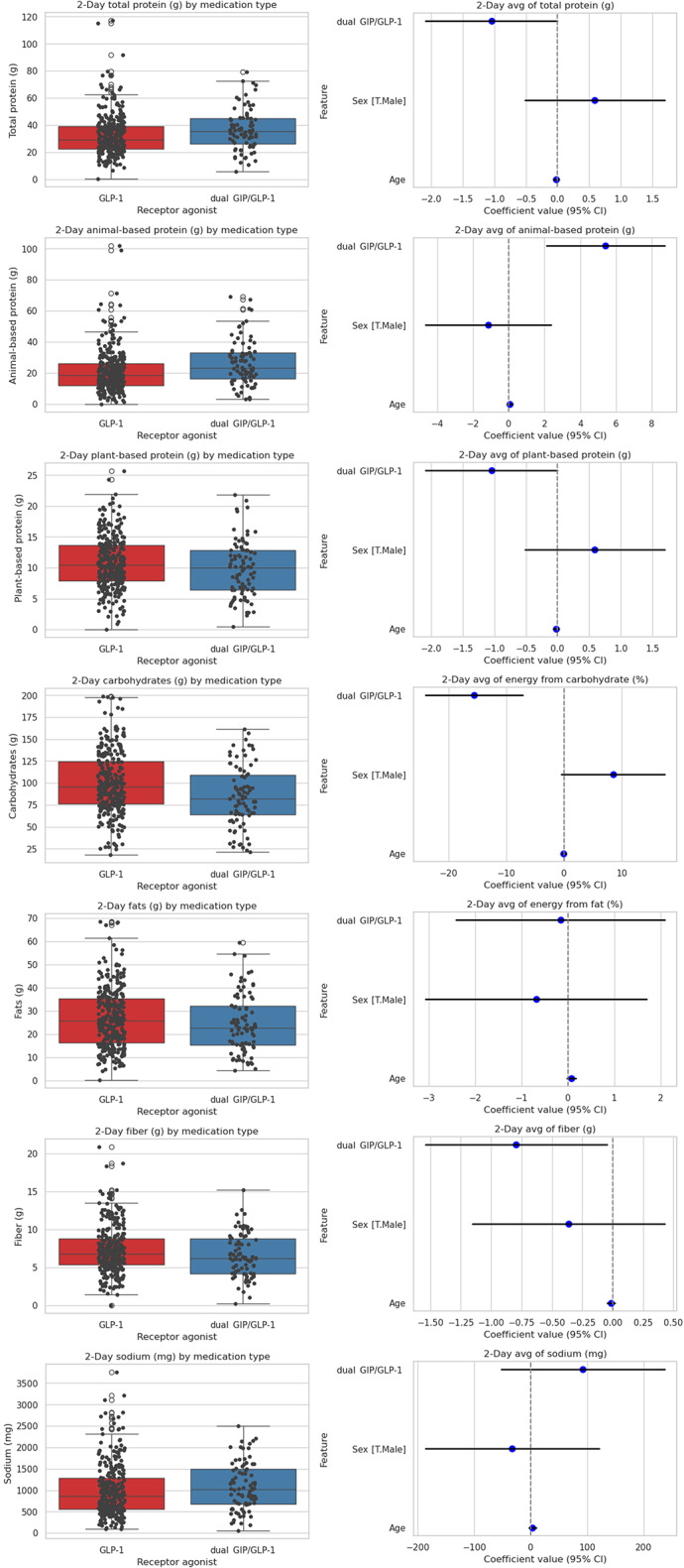
Two-day average macronutrient intake by medication class (GLP-1 RA vs. dual GIP/GLP-1) with age- and sex-adjusted differences. Left panels: distribution of 2-day mean intakes. Right panels: regression coefficients (dual GIP/GLP-1 vs. GLP-1) with 95% CIs; positive values indicate higher intake with dual GIP/GLP-1 (*n* = 387). RA, receptor agonist

Figure 4 shows the two-day average intake of macronutrients among participants receiving GLP-1 receptor agonist therapy versus dual GIP/GLP-1 therapy. Total protein intake was higher among those on dual GIP/GLP-1 compared with GLP-1 alone (mean difference: 4.29 g/day; 95% CI: 0.6, 7.9; *P* = 0.023). A similar difference was observed for animal-based protein, with participants on dual therapy consuming 5.42 g/day more (95% CI: 2.1, 8.7; *P* = 0.001). In contrast, plant-based protein intake did not differ significantly between groups, although regression analyses suggested a small decrease in the dual therapy group (–1.0 g/day; 95% CI: − 2.1, − 0.004; *P* = 0.049). Carbohydrate intake was significantly lower in the dual GIP/GLP-1 group, averaging ~ 16 g/day less than the GLP-1 group (95% CI: − 24.0, − 7.1; *P* < 0.001). Fiber intake was also slightly lower in the dual therapy group (–0.8 g/day; 95% CI: − 1.5, − 0.05; *P* = 0.037). No significant differences were found between groups for total fat or sodium intake (all *P* > 0.16).

These adjusted estimates (linear models controlling for age and sex) were directionally consistent with unadjusted independent-samples *t* tests and Mann–Whitney *U* tests (e.g., total protein: t=–2.309, *p* = 0.022; U = 10374, *p* = 0.005; animal-based protein: t=–3.055, *p* = 0.003; U = 9735.5, *p* < 0.001), supporting the conclusion that participants receiving dual GIP/GLP-1 therapy consumed more total and animal-based protein but less carbohydrate and slightly less fiber than those treated with GLP-1 receptor agonists alone.

### Predictors of weight reduction from multivariable models

**Fig. 5:**
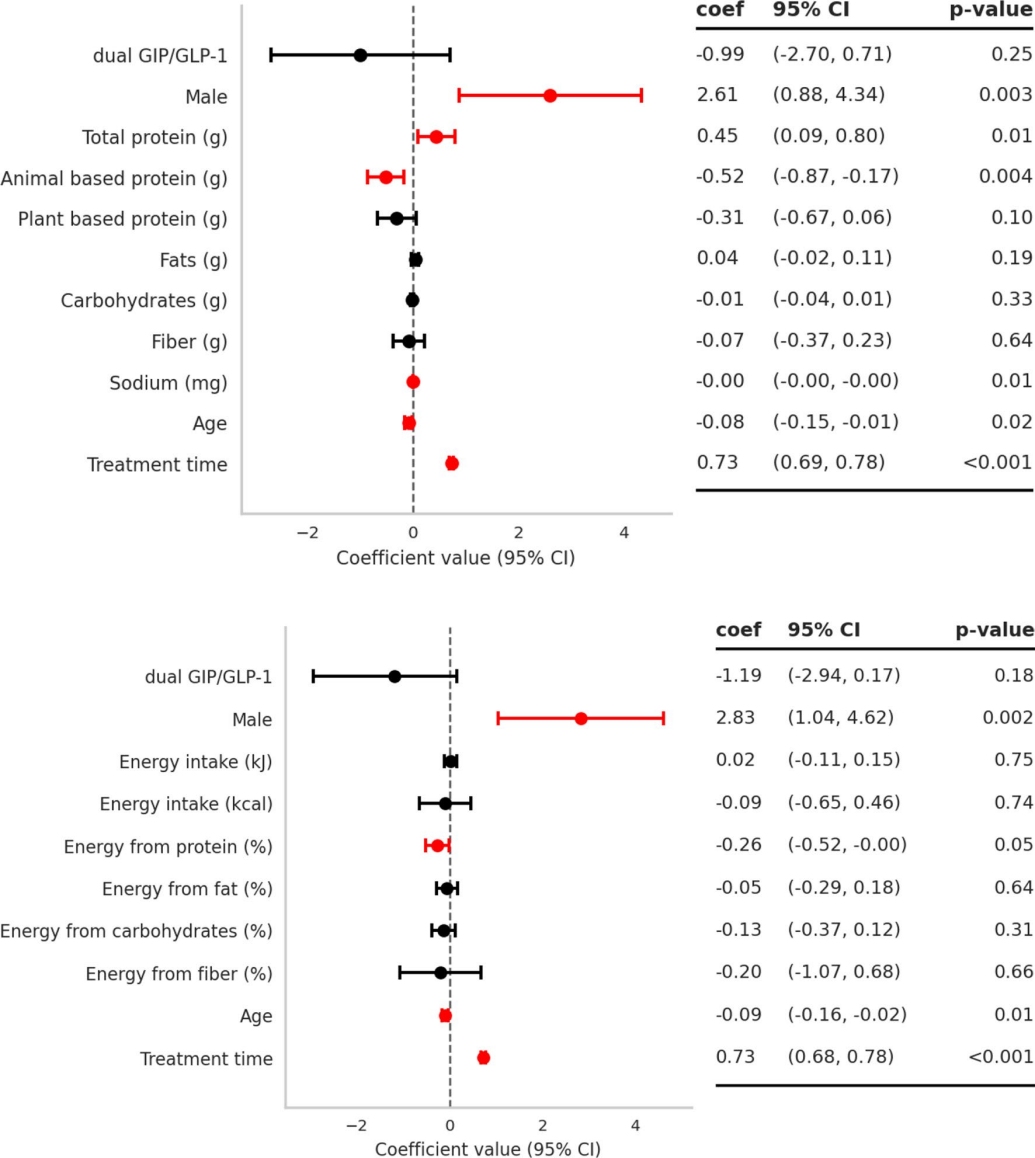
Multivariable predictors of weight reduction: models with macronutrient intake and energy distribution. Points show regression coefficients (β) with 95% CIs for associations with total weight loss (kg); positive β indicates greater loss. Covariates include drug class (dual GIP/GLP-1 vs. GLP-1), diet variables, age, sex, and treatment duration (*n* = 387)

In multivariable models, treatment duration was the dominant predictor of greater weight loss (Fig. 5). Treatment duration was the strongest predictor: each additional week was associated with ~ 0.73 kg greater weight reduction (β = 0.73; 95% CI: 0.68, 0.78; *P* < 0.001). Male participants lost more weight than females (β = 2.61; 95% CI: 0.88, 4.34; *P* = 0.003). Older age was associated with smaller reductions (β = − 0.08; 95% CI: − 0.15, − 0.01; *P* = 0.018).

Dietary factors were also related to weight outcomes. Higher total protein intake was positively associated with weight loss (β = 0.45; 95% CI: 0.09, 0.80; *P* = 0.014), whereas animal-based protein showed an inverse association (β = − 0.52; 95% CI: − 0.87, − 0.17; *P* = 0.004). Sodium intake was similarly associated with smaller reductions (β = − 0.002; 95% CI: − 0.00, − 0.001; *P* = 0.013), which seems to point to the consumption of highly processed foods rich in sodium.

The type of receptor agonist (GLP-1 vs. dual GIP/GLP-1) did not significantly predict weight reduction after adjustment for covariates (β = − 0.99; 95% CI: − 2.70, 0.71; *P* = 0.253).

A second model, including energy intake and macronutrient distribution, explained a similar proportion of variance (R² = 0.73). In this model, a higher percentage of energy from protein was modestly associated with smaller weight reductions (β = − 0.26; 95% CI: − 0.52, − 0.00; *P* = 0.047), while other dietary proportions (fat, carbohydrate, fiber) and total energy intake (kJ, kcal) were not significant predictors (all *P* ≥ 0.31).

Overall explained variance was high for both models (R²=0.745 and 0.734; adjusted R²=0.737 and 0.727; *p* < 0.001).

### Determinants of BMI reduction in multivariable analyses

**Fig. 6:**
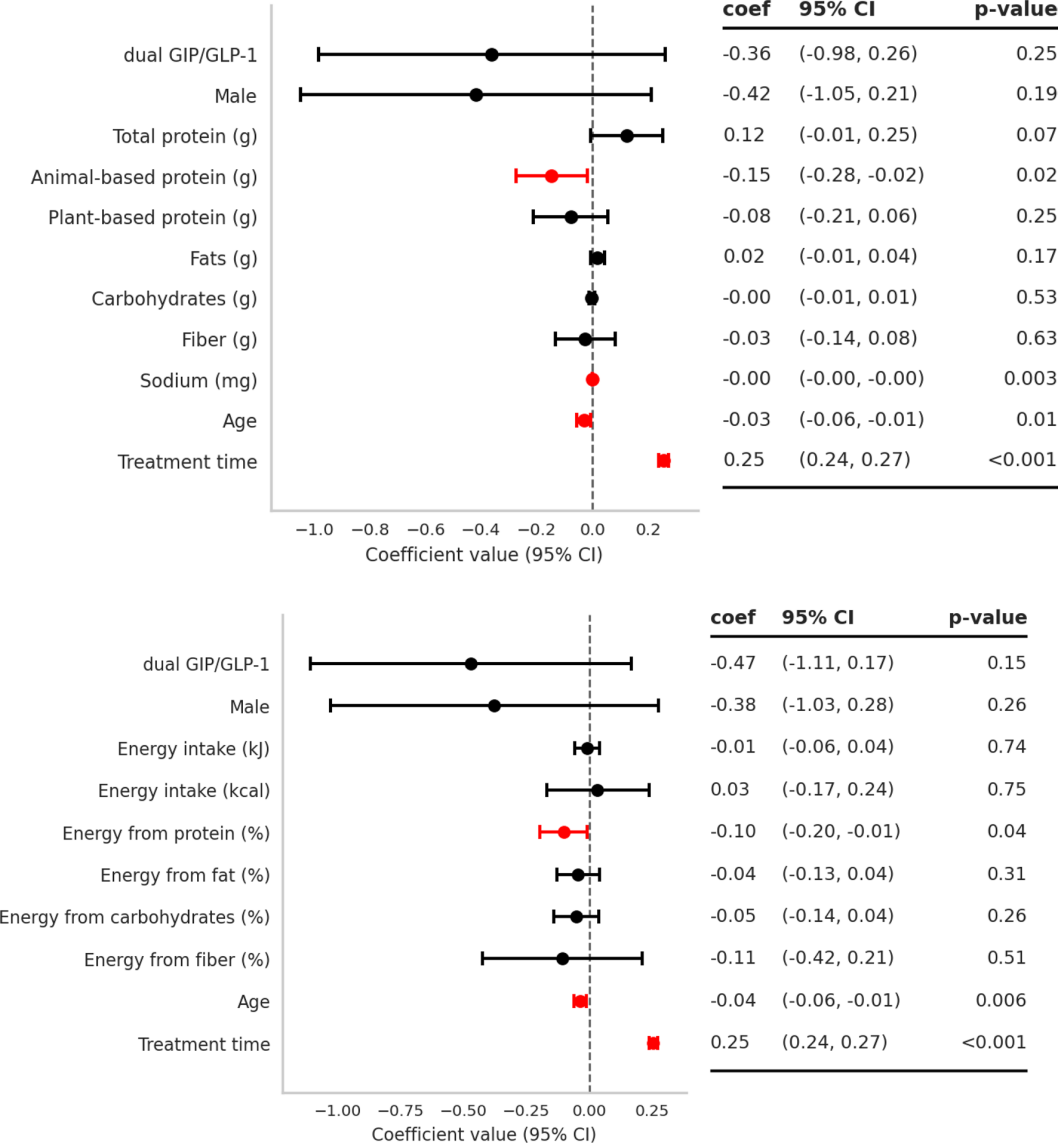
Multivariable predictors of BMI reduction: models with macronutrient amounts and energy distribution. Forest plots display regression coefficients (β) and 95% CIs for associations with total BMI reduction; positive β denotes greater reduction. Covariates include drug class (dual GIP/GLP-1 vs. GLP-1), diet variables, age, sex, and treatment duration (*n* = 387)

In adjusted models, treatment duration was the strongest predictor, with each additional week associated with ~ 0.25 units greater BMI reduction (β = 0.26; 95% CI: 0.24, 0.27; *P* < 0.001) (Fig. 6). Drug class (dual GIP/GLP-1 vs. GLP-1) and sex were not associated with BMI change after adjustment (β=–0.36 to − 0.47; *P* = 0.146–0.253; sex: β=–0.42 to − 0.38; *P* = 0.191–0.257).

Dietary variables showed modest associations. Higher sodium intake was inversely associated with BMI reduction (β = −0.002; 95% CI, − 0.003 to − 0.001; *P* = 0.003), such that greater sodium intake was related to smaller BMI decreases. Animal-based protein intake was likewise inversely associated with BMI reduction (β = −0.15; 95% CI, − 0.27 to − 0.02; *P* = 0.024), whereas total protein intake showed only a borderline positive association (β = 0.12; 95% CI, − 0.01 to 0.25; *P* = 0.066). Intakes of fat, carbohydrates, and fiber were not significantly associated with BMI change (all *P* > 0.05).

In a second model including total energy intake and macronutrient distribution, the percentage of energy derived from protein was inversely associated with BMI reduction (β = −0.10; 95% CI, − 0.19 to − 0.01; *P* = 0.036), indicating that a higher protein share of total energy was related to slightly smaller BMI decreases. Neither total energy intake (kJ or kcal) nor the proportions of energy from fat, carbohydrates, or fiber predicted BMI change (all *P* ≥ 0.26). Model fit was high for both specifications (R² = 0.720 and 0.707; adjusted R² = 0.712 and 0.699; *P* < 0.001).

## Discussion

In our study, we demonstrated that the diet of individuals taking incretin medications is inadequate and may cause complications resulting from deficiencies in nutrients that are essential for therapy.

We have shown that the diet of our respondents was characterized by too low and inadequate intake of energy, protein, and key micronutrients compared to the recommended standards. We also noted differences in energy intake between weekdays and weekends.

The results of this study (retrospective cohort study) provide valuable information on the impact of treatment with GLP-1 receptor agonists and dual GIP/GLP-1 agonists on diet composition, calorie intake, and weight loss efficacy. The observed changes in dietary patterns and the identification of key dietary factors associated with weight loss shed light on mechanisms that may modulate the metabolic response in patients undergoing obesity pharmacotherapy. The problem of insufficient dietary support for patients has been recognized—the American College of Lifestyle Medicine, the American Society for Nutrition, the Obesity Medicine Association, and the Obesity Society recently published recommendations for nutritional support in GLP-1 therapy for obesity, indicating that although these drugs are remarkably effective, there are real challenges that require comprehensive nutritional support and lifestyle changes.

In a study group of 387 adults treated with GLP-1 agonists or dual GIP/GLP-1 agonists, very low energy intake (753 kcal/day) and protein intake (33.4 g/day) were observed; less than 10% of participants met the recommendations for protein, fiber, vitamin D, calcium, and potassium intake. In addition, on weekdays, 170 kcal and 8–9 g of fat were consumed more than on weekends, indicating a rhythmic pattern increased calorie intake during the week.

To date, studies on the diet of patients taking incretin have been few and limited mainly to energy analysis [3, 24]. Our results confirm previously observed energy deficits but document for the first time such profound protein and micronutrient deficiencies during pharmacotherapy, as well as differences between weekdays and weekends [3]. These findings are of significant clinical relevance, given that substantial calorie deficits, while effective for short-term fat loss, pose a risk of nutritional deficiencies, metabolic disorders, and adverse side effects such as sarcopenic obesity [8, 10, 23]. Sarcopenic obesity, defined as the coexistence of excessive obesity and reduced skeletal muscle mass and function, is particularly concerning [3]. This condition has serious consequences, including reduced physical performance, increased risk of falls and fractures, poorer quality of life, insulin resistance, and increased cardiovascular risk. Muscle loss during weight reduction is well documented, and without appropriate interventions, up to 25–30% of lost body weight may come from lean muscle tissue. In the context of GLP-1 therapy, intense calorie restriction, if not adequately balanced with adequate protein intake and physical activity, may exacerbate muscle loss. Patients treated with GLP-1 analogues may be at increased risk due to the marked appetite-suppressing effect of these drugs, which may reduce protein intake below recommended levels [3, 24, 25]. The effect of protein quality is consistent with meta-analyses indicating that a higher proportion of plant protein correlates with greater weight loss and less loss of lean body mass [4, 5]. The unexpected negative impact of animal protein may reflect the simultaneous negative impact of consuming highly processed foods that contain not only protein but also large amounts of sodium and saturated fats.

Given the observed average intake of only 33.4 g of protein/day and 7.2 g of fiber/day, routine dietary intervention to ensure adequate protein and micronutrient intake should be standard care before and during incretin therapy. Recommendations may include: (1) achieving a target protein intake of ≥ 1.2 g protein/kg of target body weight (approximately 75–90 g/day for most patients), (2) supplementation with key micronutrients, including vitamin D, calcium, and potassium, (3) a meal plan that limits fluctuations between days of the week, individualized meal planning to minimize fluctuations in diet between weekdays and weekends, and (4) regular monitoring of sodium intake. This comprehensive nutritional approach may reduce the risk of sarcopenia, loss of lean body mass, functional impairment, and weight regain after discontinuation of pharmacotherapy.

Multiple regression analysis showed that the strongest predictor of weight loss was longer duration of treatment rather than specific type of therapy (GLP-1 vs. dual GIP/GLP-1 therapy GLP-1), which is consistent with observations indicating that the weakening of the effect of GLP-1 analogues over time may be caused, for example, by a reduction in the effect on satiety centers. The result supports the use of long-term pharmacological, dietary, and behavioral interventions in treatment. Interestingly, while higher total protein intake was positively correlated with successful weight loss, animal protein showed a negative correlation. This finding suggests that the source of protein (from highly processed foods) has a significant impact on weight control outcomes, possibly due to differences in bioavailability, thermic effect, sodium content, or impact on gut microbiota, which requires further investigation [4, 5]. 

The significantly lower carbohydrate and fiber intake observed in patients treated with dual GIP/GLP-1 therapy, despite higher protein intake, indicates potential deficiencies in diet quality that may impact metabolic health. Dietary fiber plays a well-known role in controlling blood sugar levels and maintaining a healthy weight, which highlights the need to monitor the dietary intake of this component as a supplement to pharmacological treatment.

Low energy and nutrient intake across the group, particularly protein, fiber, and key micronutrients, highlights the importance of closely monitoring the nutritional status of patients receiving GLP-1 therapy. This monitoring is critical to minimizing the risk of complications associated with malnutrition. It is recommended that pharmacological care be combined with intensive dietary support and patient education, especially at the start and during incretin-based therapy. Identified nutritional deficiencies should be monitored individually and addressed through multidisciplinary interventions involving physicians and dietitians to optimize treatment outcomes, patient functioning, and overall quality of life.

Further prospective studies involving objective assessment of body composition and monitoring of physical activity are needed to establish optimal nutritional guidelines to support long-term incretin therapy and prevent sarcopenic obesity [3, 11, 24–26].

Our study had both strengths and weaknesses. A key strength is the comprehensive evaluation of macronutrient intake and anthropometric outcomes in relation to GLP-1 and dual GIP/GLP-1 receptor agonist therapies in a substantial cohort of adults with obesity (*N* = 387). The dietary assessment captured two consecutive days, one weekday and one weekend day, allowing nuanced comparisons of routine and leisure-period intake. High-quality data collection procedures, including participant-driven validation of dietary records, further strengthen reliability and support broader generalizability. In addition, the use of complementary analytic frameworks, classical frequentist statistics alongside Bayesian approaches, provides convergent evidence and richer inference about dietary intake and body-composition changes during pharmacotherapy for obesity.

Important limitations should also be noted. Dietary intake was self-reported via food diaries and is therefore vulnerable to recall bias and under- or over-reporting, particularly among individuals with obesity. Additionally, dietary intake was recorded only over two days, limiting the ability to generalize dietary patterns beyond this short time-frame. Despite rigorous statistical adjustments, the observational design precludes definitive causal inferences about the relationships observed. Furthermore, participant recruitment through social media and internet forums may introduce selection bias, potentially limiting the representativeness of the general population with obesity. The lack of long-term follow-up beyond the end of treatment constrains our understanding of sustained dietary behavior changes and weight management outcomes.

In conclusion adults receiving incretin pharmacotherapy were found to have very low energy, protein, and key micronutrient intakes and marked differences between weekdays and weekends. The integration of dietary care aimed at adequate protein and micronutrient intake should accompany GLP-1/GIP-GLP-1 treatment to maximize weight loss efficacy and minimize the risk of sarcopenia.

The use of medications without proper education on nutrition during pharmacotherapy or without individual dietary consultation (if necessary) may lead to a deepening of macro- and micronutrient deficiencies.

Further prospective studies with body composition measurements and physical activity monitoring are needed to determine the optimal composition of a diet supporting incretin therapy.

## Data Availability

The datasets used and analysed during the current study are available from the corresponding author on reasonable request.

## Abbreviations

BMI: Body mass index
GLP-1: Glucagon-like peptide-1
GIP: Gastric inhibitory polypeptide
RDA: Recommended dietary Allowance
